# Observation of Ice-Like Two-Dimensional Flakes on Self-Assembled Protein Monolayer without Nanoconfinement under Ambient Conditions

**DOI:** 10.1007/s40820-025-01689-1

**Published:** 2025-03-14

**Authors:** Wuxian Peng, Linbo Li, Xiyue Bai, Ping Yi, Yu Xie, Lejia Wang, Wei Du, Tao Wang, Jian-Qiang Zhong, Yuan Li

**Affiliations:** 1 Key Laboratory of Organic Optoelectronics and Molecular Engineering, Department of Chemistry, Tsinghua University, Beijing, 100084 People’s Republic of China; 2 School of Physics, Hangzhou Normal University, Hangzhou, 311121 Zhejiang People’s Republic of China; 3 School of Materials and Chemical Engineering, Ningbo University of Technology, Ningbo, 315211 Zhejiang People’s Republic of China; 4 Institute of Functional Nano & Soft Materials (FUNSOM), Jiangsu Key Laboratory for Carbon-Based Functional Materials & Devices, Soochow University, Suzhou, 215123 Jiangsu People’s Republic of China

**Keywords:** Self-assembled monolayers, 2D ice-like water, Water–protein interactions, Ice phase transition

## Abstract

Directly correlating the morphology and composition of interfacial water is vital not only for studying water icing under critical conditions but also for understanding the role of protein–water interactions in bio-relevant systems. In this study, we present a model system to study two-dimensional (2D) water layers under ambient conditions by using self-assembled monolayers (SAMs) supporting the physisorption of the Cytochrome C (Cyt C) protein layer. We observed that the 2D island-like water layers were uniformly distributed on the SAMs as characterized by atomic force microscopy, and their composition was confirmed by nano-atomic force microscopy-infrared spectroscopy and Raman spectroscopy. In addition, these 2D flakes could grow under high-humidity conditions or melt upon the introduction of a heat source. The formation of these flakes is attributed to the activation energy for water desorption from the Cyt C being nearly twofold high than that from the SAMs. Our results provide a new and effective method for further understanding the water–protein interactions.

**Supplementary Information:**

The online version contains supplementary material available at 10.1007/s40820-025-01689-1.

## Highlights

- The two-dimensional (2D) ice-like water layers are formed on the self-assembled monolayers (SAMs), which support the physisorption of Cytochrome C (Cyt C) protein layer under ambient conditions, without the need for nanoconfinement.
- The morphology, composition, melting, and crystallization processes of the 2D ice-like water are directly characterized using atomic force microscope and nano-atomic force microscopy-infrared spectroscopy.
- The formation of the 2D ice-like water is attributed to the fact that the activation energy for water desorption from Cyt C is nearly twice as high as that from the SAMs.

**Supplementary Information:**

The online version contains supplementary material available at 10.1007/s40820-025-01689-1.

## Graphical abstract

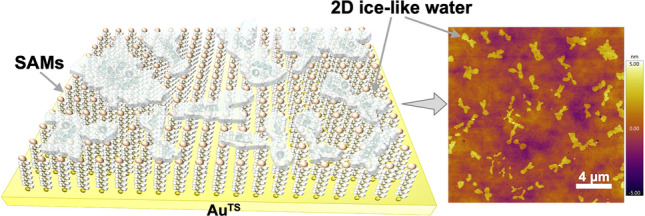

## Introduction

The interfacial water on proteins plays key roles in proteins’ stabilities, dynamics and functionalities in a wide variety of biosystems [1–6]. These roles range from preventing protein collapse [7] to participating in processes such as ligand binding [8] and folding [9], lubrication between proteins and lipids [10], and exchange of hydrogen atoms of the amide backbone with the surrounded solvent [11]. Although it has been demonstrated that the hydration layer on proteins can resemble ice-like water [12], the precise structure of this ice-like water and the mechanisms by which interactions that proteins can induce ice formation remain poorly understood. One of the main challenges is the simultaneous characterization of the topography and composition of protein–water complexes, particularly under normal pressure and temperature (NPT) conditions without nanoscale confinement. Therefore, there is an urgent need to develop methods for stabilizing ice-like water on proteins under these conditions to address outstanding questions regarding protein–water interactions.

Here, we developed a method to utilize self-assembled monolayers (SAMs) of sodium 11-mercaptoundecane-1-sulfonate (HSC_11_SO_3_Na) assembled on an ultra-flat template-stripped gold (Au^TS^) surface as the substrate, to achieve physisorption and then partially desorption of Cytochrome C (Cyt C) [13–16], at ambient condition. Using atomic force microscopy (AFM), we directly imaged island-like ice plateaus on the surfaces, revealing their unexpected growth under high humidity or melting upon heating via continuous AFM scanning. Complementary characterization techniques, including nano-atomic force microscopy-infrared spectroscopy (AFM-IR), Fourier transform infrared (FTIR) reflectance measurements, and confocal Raman spectroscopy, confirmed the composition of these ice-like plateaus. Temperature-programmed infrared reflection absorption spectroscopy (IRRAS) measurements revealed a nearly 2 times stronger interaction between water and Cyt C than the SAMs. This work sheds new light on the formation of the 2D ice-like flakes under NPT conditions without nanoscale confinement, contributing to the further understanding of the unique interactions between interfacial water and proteins.

The specific characterizations of the ice-like water have been extensively investigated [17–22], with key findings covering the following four aspects: (1) Water molecules adsorbed at the interfaces are prone to arrange into a hexagonal hydrogen-bond network, with the oxygen atoms lying on two different heights, similar to the basal plane of hexagonal ice, and are referred to as “ice-like water” or “ice bilayer” [23]; (2) in contrast to the bulk ice, which follows the Bernal–Fowler–Pauling ice rules, the 2D ice-like water exhibits distinct properties, including non-tetrahedral bonding geometries and anomalous self-diffusion [24]; (3) various crystalline structures of ice-like water have been identified, including square, rhombic, hexatic, and superionic ice phases, as well as the amorphous structures [25–27]; (4) ice-like water is verified to possess anomalously high melting temperature, suggesting its potential stability even under ambient environment [23]. These unique features of ice-like water enable it to control the physical, chemical, and biological properties of interfaces in variety of processes, such as low dielectric constant [28], near-frictionless behavior [29, 30], high electrical conductivity [31], surface adhesion [32], protein-specific recognition [8], and maintaining the activity of the proteins [33, 34]. While previous studies on ice-like water have typically been conducted under strong nanoconfinement, either high pressure or low temperature, the preparations of the ice-like water under ambient conditions without nanoscale confinement would be advantageous for further exploring its formation mechanisms and protein–water interactions. Recently, it has been theoretically proposed that the liquid-to-ice transition could occur without nanoscale confinement at room temperature, provided that the water–interface interaction is sufficiently strong to compensate for the entropy loss associated with the phase transition [35]. This opens up the possibility of directly observing 2D ice on surface.

Despite the critical role of interfacial water in maintaining the structural and functional integrity of proteins, the detailed mechanisms underlying protein–water interactions remain obscured in complex cellular and biological systems. SAMs offer a more flexible and biologically relevant environment [36] compared to commonly investigated surfaces like metals [23, 37], insulating materials [38] or graphite [27, 39]. Analyzing the properties of protein–water interfaces on SAMs can thus provide valuable insights into a wide range of biologically related biomechanics and physicochemical processes, including protein folding, ligand interactions of proteins, and drug transport.

## Experimental Section

### Preparation of Au^TS^ Substrates

The Au^TS^ substrates were fabricated by the procedures as our previous work reported [40, 41]. Briefly speaking, the Au film (Au with a purity of 99.999% from Dimu Materials, Inc (China)) with thickness of 200 nm was deposited on the clean Si (100) wafers with a native SiO_2_ passive film via thermal deposition (KYKY-400, Zhongke Ke Yi, China) with the evaporation rate was about 0.2 Å s^−1^ at the first 50 nm and then increased to ~ 1 Å s^−1^ for the rest 150 nm and the base pressure being 2 × 10^–5^ Pa. The glass slides (1 × 1 cm^2^) were ultrasonic-cleaned with acetone and ethanol for about 20 min. Then a stream of N_2_ gas was used to dry the slides. Then a plasma of air was used to clean the slides for 5 min at a pressure of 100 Pa. The photo-curable optical adhesive (Norland, No. 61) was utilized to glue the glass slides on the Au surface. To cure the optical adhesive, the Au substrates with glass slides were placed under a 100-Watt UV lamp. After that, the Au surface can be lift off by a razor blade, which had been in touch with the Si/SiO_2_ wafer [40].

### Preparation of Self-Assembled Monolayers (SAMs)

The synthetic processes of sodium 11-mercaptoundecane-1-sulfonate (HSC_11_SO_3_Na) have been reported in our recent work [42]. In order to form SAMs, the Au substrate was immersed in degassed 1 mM ethanolic solutions of HSC_11_SO_3_Na for a period of time over 12 h in an inert nitrogen environment. Then, to remove the physisorbed molecules, the chips were rinsed with AR grade ethanol and gently dried in a nitrogen stream. After that, the SAMs supported by Au^TS^ substrate were immersed in phosphate-buffered saline (PBS, 1X, pH 7.2–7.4, Adamas life®) solution containing Cytochrome C (Cyt C) (1 mg mL^−1^) for 30 mins to allow the physisorption of the protein. Finally, the chips were removed from the solution and continuously rinsed with the PBS solution, followed by drying the surface with mild N_2_ gas flow to partially desorb the Cyt C.

### AFM Characterization of the Ice-Like 2D Flakes

The AFM images were recorded by the Cypher Oxford Instrument with tapping mode tips (AC200TS-R3, resonant frequency: 150 kHz, force constant: 200 N m^−1^). It should be noted that the detection of the ice-like water in this work must be under the Asylum’s blueDrive excitation technique, which takes advantage of a laser to directly excite the cantilever resonance without the detection laser inducing the undesired temperature enhancement. This technique uses a laser, which is focused on the base of the cantilever, to replace the tapping piezo.

### Nano-AFM-IR Characterization of the Ice-Like 2D Flakes

Nano-IR2 from Anasys Instruments (now Bruker Corporation)) with a standard Anasys contact mode was used to interrogate the chemical analysis and compositional mapping of the island-like plateaus at the nanoscale resolution of ~ 20 nm. Nano-infrared uses photothermally induced resonance technique (AFM-IR) for microchemical analysis to provide nanoscale spectra and composition distribution. The infrared beam shines on the sample, the sample absorbs the radiation wave of a specific wavelength, and the heat generated by the absorption of the radiation causes rapid thermal expansion of the sample, which causes the micro-cantilever of the AFM probe to produce resonance oscillation. The local infrared absorption spectrum can be obtained using the Fourier transform to extract the amplitude signal of resonance oscillation and establish its relationship with the wavelength of the light source. The sample is irradiated by pulsed infrared with a fixed wavelength, and the infrared absorption information of the sample under the wavelength is collected by a probe, and infrared absorption imaging is carried out to reflect the distribution information of the chemical groups represented by the wavelength on the sample surface.

### Infrared Reflection Absorption Spectroscopy (IRRAS)

In situ IRRAS experiments were carried out in a home-built ultrahigh-vacuum (UHV) system with a base pressure better than 2 × 10^–10^ torr [43]. The UHV system consists of a preparation chamber for sample cleaning and preparation and a measurement chamber for the IRRAS experiments. The measurement chamber is equipped with an FTIR spectrometer ((Bruker VERTEX 70v) connected by KBr windows. The IR beam is reflected from the sample surface plane at an angle of 7°, and the signal is measured with a liquid N_2_-cooled MCT detector. The SAMs or the Cyt C/SAMs sample was mounted on a Mo sample holder using Ta strips and was heated by resistive heating and cooled with liquid N_2_. The sample temperature was measured by a chromel–alumel thermocouple placed at the edge of the sample. The sample was degassed at 350 K for at least 3–5 h before water deposition experiments. High-purity D_2_O (from Aldrich, > 99.96% D, or from Energy Chemical, > 99.9% D) and H_2_O (from Macklin, HPLC) were used in the experiments, which were further purified under vacuum by several freeze–thaw cycles. Two independent gas lines were used to introduce the D_2_O and H_2_O into the measurement chamber, and the purity of the water was checked in the preparation chamber by a quadrupole mass spectrometer (Hiden HAL 3F/PIC). The water molecules were deposited in situ onto the sample surface kept at ~ 110–115 K through a leak valve. The exposure (or the thickness of the water films) was quoted in Langmuir (1 L = 1 × 10^6^ torr s). The IRRAS spectra were recorded with *p*- and *s*-polarized light with 256 scans and a spectral resolution of 4 cm^−1^. During the temperature-programmed measurements, the temperature of the sample was ramped up at a rate of 0.01 K s^−1^, and the IRRAS spectra were continuously acquired at a rate of 62 s per spectrum.

## Results and Discussion

### Preparation Processes of the Ice-Like Water

**Fig. 1:**
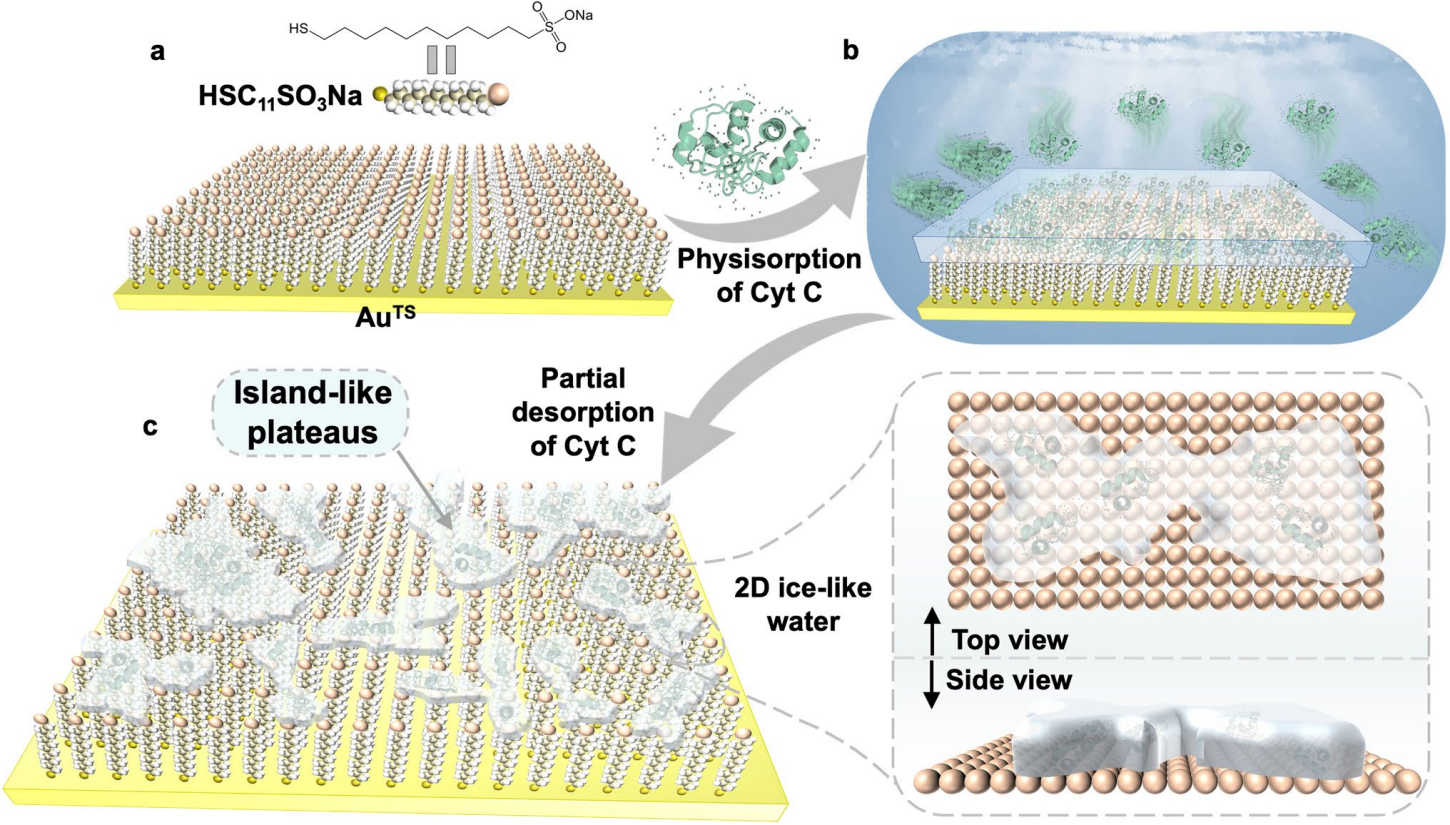
Schematic diagram of the preparation processes of the ice-like water layers, including **a** SAMs of HSC_11_SO_3_Na anchored on the Au^TS^ substrate; **b** Cyt C adsorbed on the SAMs in PBS solution. **c** Observation of the island-like plateaus after partial desorption of Cyt C. Top and side view of the atomic model of 2D ice-like water on SAMs [49]

**Fig. 2:**
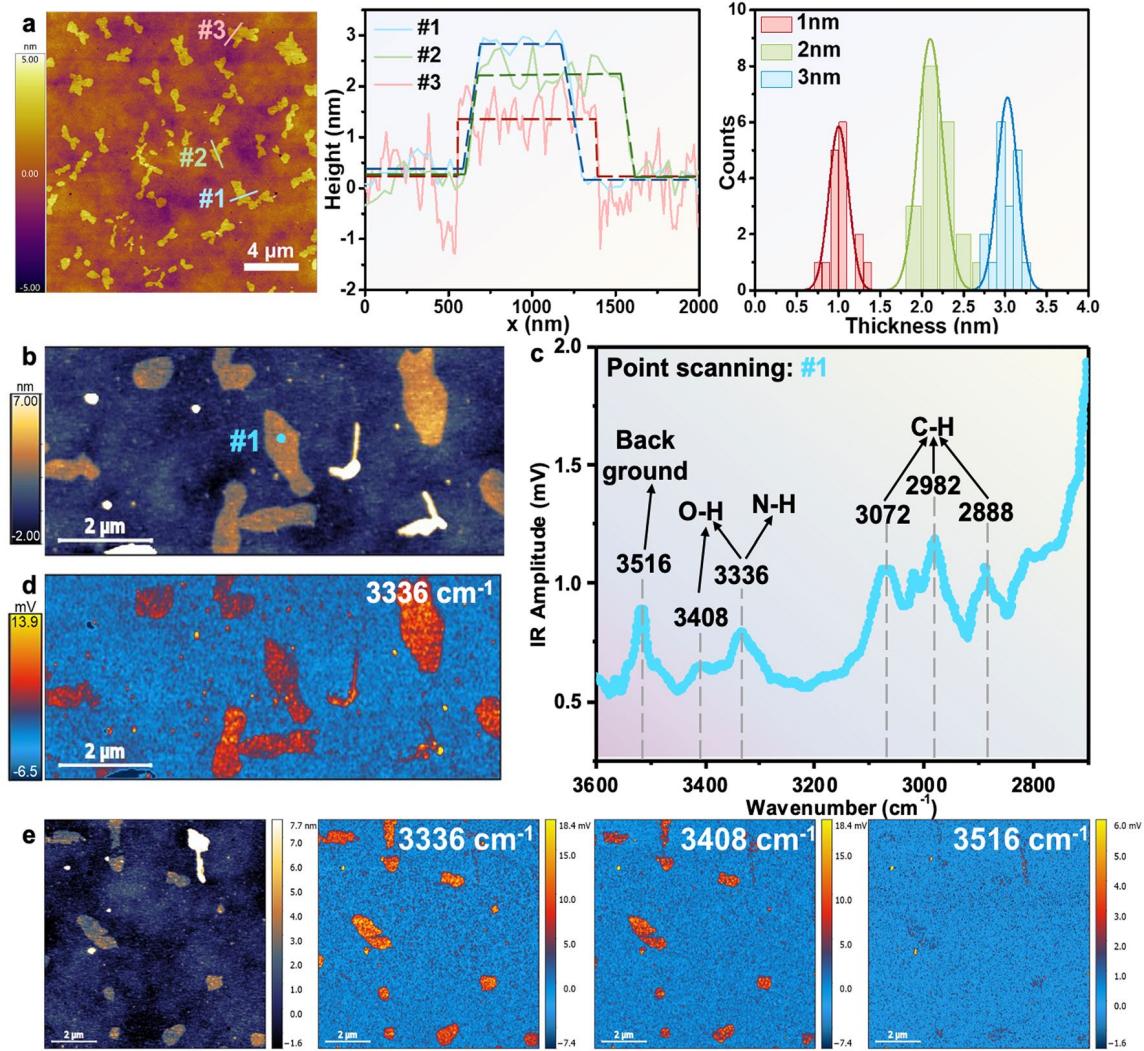
**a** Typical AFM image of island-like plateaus on the surface of SAMs (room temperature, RH ~ 60%), the corresponding height line profiles, the dashed lines are the guides to eyes and the histogram illustrating the height distribution of the 2D flakes. **b** AFM-IR height image. **c** AFM-IR spectra of spot #1 in panel **b**. **d** AFM-IR absorption image recorded at 3336 cm^−1^ that corresponded to the distribution of the ice-like water. **e** AFM-IR height image and the related nano-IR images were recorded at 3336, 3408, and 3516 cm^−1^, respectively. The AFM-IR characterizations were carried out at room temperature, RH ~ 60%

Figure 1 schematically shows the preparation process for generating the ice-like water, which can be divided into three steps. Firstly, the SAMs of HSC_11_SO_3_Na were assembled on the Au^TS^ substrate (Fig. 1a), which is an ultra-flat Au^TS^ substrate with root-mean-square (RMS) roughness less than 0.5 nm [40, 41]. The synthetic methods of HSC_11_SO_3_Na, and the preparation of the SAMs were previously reported by us and were summarized in Section S2 [42]. Studies from our and other groups [44–47] have confirmed that these molecules can anchor on the Au^TS^ substrates via S–Au covalent interactions [48], forming densely packed monolayers with a smooth surface and a thickness of approximately one molecular length. A flat surface is crucial for the growth of ice-like water, as corrugated gold surfaces are undesirable for the formation of robust ice layers [23]. Secondly, the physisorption process of Cyt C protein molecules onto the SAMs occurred (Fig. 1b). We immersed the SAMs supported by Au^TS^ chips in phosphate-buffered saline (PBS, 1X, pH 7.2–7.4, Adamas life®) solution containing Cyt C (1 mg mL^−1^) for 30 min. The electrostatic interactions between the negatively charged sulfonate functional groups of the SAMs and the positively charged amino acids close to the heme group of Cyt C facilitated the adsorption of the Cyt C layer on top of the SAMs [13, 14]. Thirdly, the samples were removed from the solution and continuously rinsed with the PBS solution, followed by drying the surface with mild N_2_ gas flow to partially desorb the Cyt C (Fig. 1c). Subsequently, the island-like plateaus with evenly distributed and irregularly shaped ice-like water layers, ranging in thickness from ~ 0.7 to ~ 3.3 nm based on the statistical results in Fig. 2a and composed of a combination of ice-like water and protein, were directly visualized on the SAMs using AFM and nano-AFM-IR characterizations. The schematic atomic model of the structured water layers is also presented in Fig. 1c, based on our observations shown in Fig. 2 and the reported works of other groups [18, 23]. Notably, the entire processes were carried out at NPT conditions.

### Topography and Composition Characterizations for the Ice-Like Water

The topographies of the 2D plateaus on the SAMs were imaged using the Cypher VRS AFM (Fig. 2a), employing the Asylum’s blueDrive excitation technique, which utilizes the blueDrive laser focused on the base of the cantilever. This is very important as it avoids the undesired rapid temperature increase that may occur when the detection laser is focused on the AFM tip. The surface morphology of the Au^TS^ substrate (Fig. S1a) contained clear grain boundaries with grain sizes generally smaller than 100 nm, and the surface was relatively flat with an RMS roughness of 0.48 nm. After the SAMs of HSC_11_SO_3_Na were anchored on the Au^TS^ substrate, the grain boundaries became blurred, and the surface roughness (RMS = 0.45 nm) was similar to the bare Au^TS^ substrate (Fig. S1b). Upon adsorption of Cyt C onto the surface, granular proteins were detected on the SAMs with a height of 3.86 ± 0.54 nm (Figs. S1c, d and S2a). The detailed characterizations of the SAMs after Cyt C physisorption were discussed in our recent work [42], including X-ray photoelectron spectroscopy (XPS) and surface plasmon resonance. After the partial desorption of Cyt C, uniformly distributed island-like plateaus with heights ranging from ~ 0.7 to ~ 3.3 nm were visualized on the SAMs (room temperature with relative humidity (RH) of ~ 60%) as shown in Fig. 2a, which were distinct from the granular morphology of Cyt C (Fig. S2). The observed ice-like water with varying thickness may originate from the distinct localized pressure of each flake, which can be induced by the defects of the Au^TS^ substrate and the SAMs as well as the detailed adsorption states of Cyt C [5]. We also immersed the SAMs of HSC_11_SO_3_Na supported by Au^TS^ substrate in PBS without Cyt C and a solution of Cyt C (1 mg mL^−1^) in pure water for 30 min, respectively, which acted as the control groups of immersion the SAMs in PBS with Cyt C (1 mg mL^−1^). The surface morphology of the related samples was characterized by AFM (Fig. S3), and we found that there were no 2D flakes as observed in Fig. 2, which demonstrated that the physical adsorption of Cyt C in PBS solution was the necessary condition for the preparation of the 2D layers in our work (We gave a mini-discussion about this in Section S1).

Considering that the thickness of a monolayer water molecules is approximately 0.2 nm [18], the ice-like water we prepared was assumed to consist of multiple layers of water molecules, ranging from a few layers to roughly more than 10 layers. The line-scan profiles in Figs. 2a and S4-S7 revealed three key characteristics: (i) each island-like plateaus exhibited a relatively uniform height; (ii) the histogram in Fig. 2a illustrated that the thicknesses of the flakes ranged from ~ 0.7 to ~ 3.3 nm, with a concentration around 1.0, 2.0, and 3.0 nm; (iii) the height line profiles were not completely straight (Figs. S5, S6 and S7), and it appeared that the thicker the plateaus, the flatter the surface. This suggests that surface roughness tends to decrease to a certain extent as the 2D flakes grow.

Nano-AFM-IR with a standard Anasys contact mode was utilized to further interrogate the chemical analysis and compositional mapping of the island-like plateaus at the nanoscale resolution of ~ 20 nm (Figs. 2b–e and S8) [50–52]. Figure 2b shows the topography of 2D plateaus imaged by Bruker AFM similar to that of Fig. 2a imaged by Cypher VRS AFM. Figure 2c shows the measured nano-AFM-IR spectra of spot #1 in Fig. 2b. The absorptions at 2850 ~ 3100 cm^−1^ were the C-H stretching modes and 3300 ~ 3500 cm^−1^ were the O–H and N–H stretching modes [52]. It is noted that the nano-IR spectrum is recorded by the energy normalization with the laser background energy (Fig. S9), reflecting the absorption intensity per unit of energy. A sudden jump in the laser background energy around 3520 cm^−1^ will cause the unexpected absorption in the nano-IR spectrum, resulting in the false “peak” as shown in Fig. 2c. Therefore, the wavenumber of 3516 cm^−1^ was attributed to the background signal rather than the infrared absorption of the stretching vibrations of molecules on the Au^TS^ chips. Ice-like water was confirmed from the characteristic IR band at 3336 cm^−1^ with a shoulder peak at 3408 cm^−1^ as the O–H stretching modes [53–55]. It should be noted that the absorption at 3336 cm^−1^ could also correspond to the N–H stretch vibrations of the protein [56]. However, Cyt C typically maintains a folded state similar to its globular form in PBS solution [3], which is significantly distinct from the characteristic topography of the 2D plateaus. Focusing on the spectral range of 1300 ~ 1900 cm^−1^ in the nano-IR spectrum (Fig. S10 and Table S1), we observed the characteristic amide I band at 1650 cm^−1^ and amide II band at 1542 cm^−1^ of the protein [57]. In addition, Fourier transform infrared (FTIR) reflectance measurements were conducted (see Section S2 for details). The wavenumber at 1651 and 1543 cm^−1^ correspond to the typical amide I (C = O) and amide II (C–N) stretching mode, respectively, while the IR band at 3359 cm^−1^ is attributed to the O–H stretching (Fig. S11, Table S1) [57]. Based on these findings, we propose that the observed flakes are mainly composed of water and adsorbed Cyt C.

The high-resolution IR mapping was collected at 3336 cm^−1^ to compare the topography within the sample (Fig. 2b) with the localized chemical domains based on color intensity (Fig. 2d), where the yellow/red color indicated strong absorption and the blue/black color indicated weak or no absorption. The distribution of areas with strong absorption of 3336 cm^−1^ in the nano-IR image (Fig. 2d) was consistent with the morphology of the island-like plateaus in Fig. 2b, providing further evidence of the formation of 2D ice-like water on the SAMs. The AFM-IR absorption maps with IR source adjusted to 3336, 3408, and 3516 cm^−1^, respectively, were collected in the same area of the sample (Fig. 2e). Both the mappings at 3336 and 3408 cm^−1^ were nearly coincident with the morphology of uniformly distributed island-like plateaus, while the mapping recorded at 3516 cm^−1^ displayed almost no absorption on chips. The chemical analysis and compositional imaging of island-like plateaus by nano-AFM-IR characterizations proved solid evidence of the existence of 2D ice-like water on the SAMs with good thermostability at NPT without nanoscale confinement. Additionally, confocal Raman spectroscopy was used to evaluate the vibrational information of the surface (Fig. S12). We observed a sharp near triangle-shaped peak at 3390 cm^−1^, the characteristic peak that is the Raman signal of ice [58, 59], which further identifies the presence of water on the SAMs after partial desorption of the Cyt C (see Section S3 for details).

### Melting and Crystallization Processes of the Ice-Like Water

**Fig. 3:**
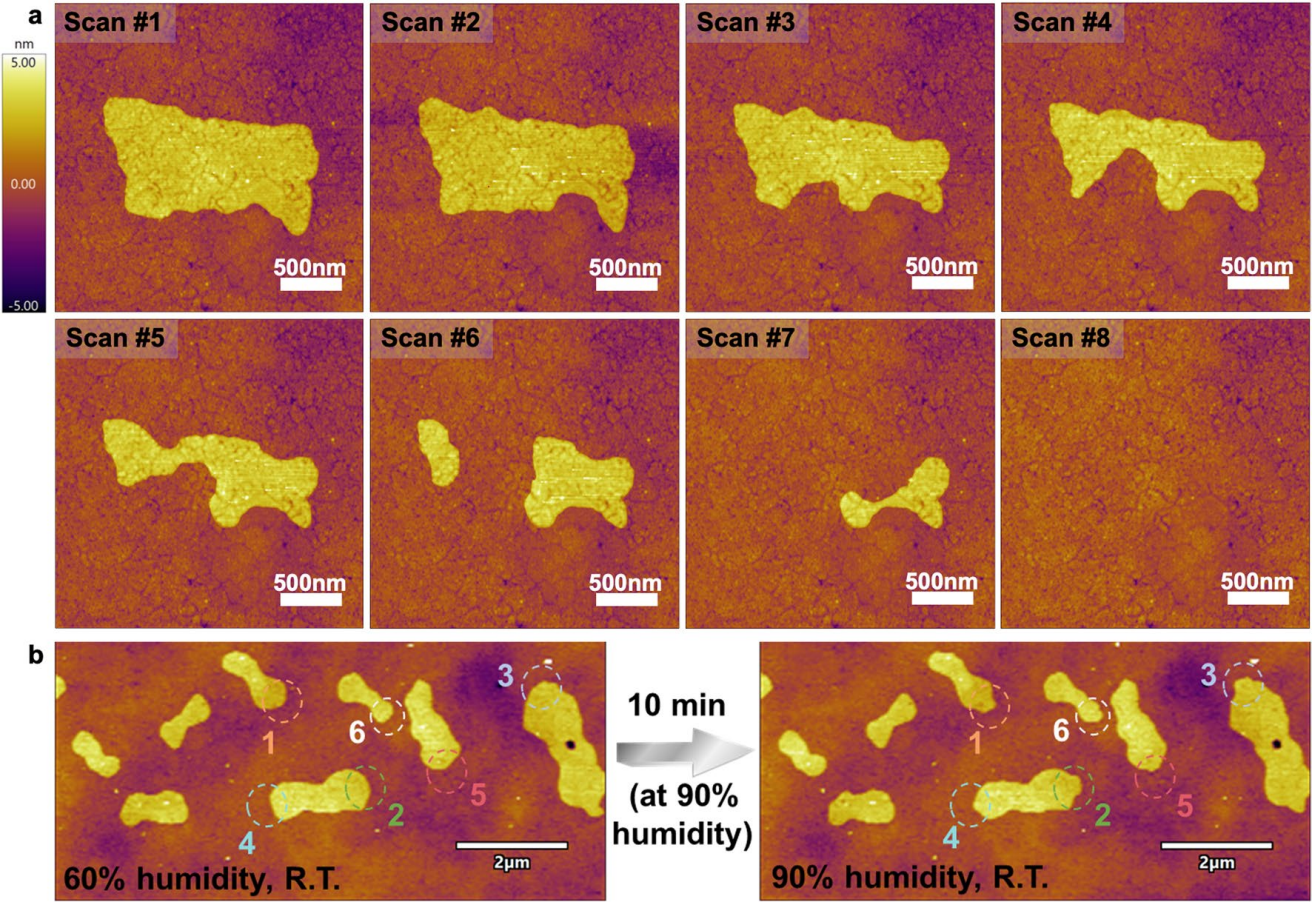
**a** Melting followed by the evaporation process of the island-like plateaus by continuous scanning of the AFM tip (room temperature, RH ~ 60%). **b** Growth of the island-like plateaus is achieved by increasing the RH from ~ 60% to ~ 90% and turning off the light source inside the AFM chamber for 10 min. The growing areas were circled with dashed circles

To gain insight into whether the 2D flakes exhibit typical ice behaviors, such as melting and crystallization processes, we continuously collected AFM images in the AC mode at a scan rate of 9.77 Hz, scanlines of 256 and scanpoints of 512, as shown in Fig. 3. We analyzed how the localized temperature enhancement induced by the consecutive scanning affects the topography of the 2D flakes. Surprisingly, the surface coverage of island-like plateaus gradually shrunk and eventually disappeared completely because of the continuous scanning of the AFM tip (Figs. 3a and S13, S14). The whole process was very similar to the melting of ice [60], and the reasons for this can be attributed to the constant heating of the tip by the blueDrive laser during AFM continuous scanning, leading to a sharp increase in the local temperature at the tip area. A movie recording the dynamic disappearance processes of the plateaus is provided in Video S1, where the left and right panels are recorded from the height sensor channel and the phase channel, respectively. It is inferred that the evaporation process could immediately occur after the melting of ice, given the fact that we did not observe any “liquid” left on the surface after heating the samples. This melting, followed by immediate evaporation behavior, further indicates the island-like plateaus mainly consist of the 2D ice-like water with good thermal stability under NPT.

The mechanism underlying the construction of the ice-like water on the SAMs may come from the electrostatic interactions between the sulfonate group at the surface of SAMs and the surface of Cyt C, forming a hydration layer fused into the 2D flakes [29]. This hydration layer dramatically improved the interaction between the water–SAMs interface, which was sufficient to compensate for the entropy reduction of the liquid–solid phase transition of interfacial water [35].

Additionally, the melting of ice-like water was observed during continuous scanning of nano-AFM-IR as shown in Fig. S15 marked with white dashed blocks. Interestingly, we also found that the edges of some island-like plateaus could grow outward, as circled by dashed circles in Fig. 3b, after increasing RH from 60% to 90% inside the AFM chamber over 10 min. Therefore, we have directly observed 2D ice-like water on the SAMs of HSC_11_SO_3_Na, and the morphology of the 2D flasks can be manipulated by temperature and relative humidity under room temperature and without nanoconfinement.

### Activation Energy for Water Desorption Derived from In Situ IRRAS

**Fig. 4:**
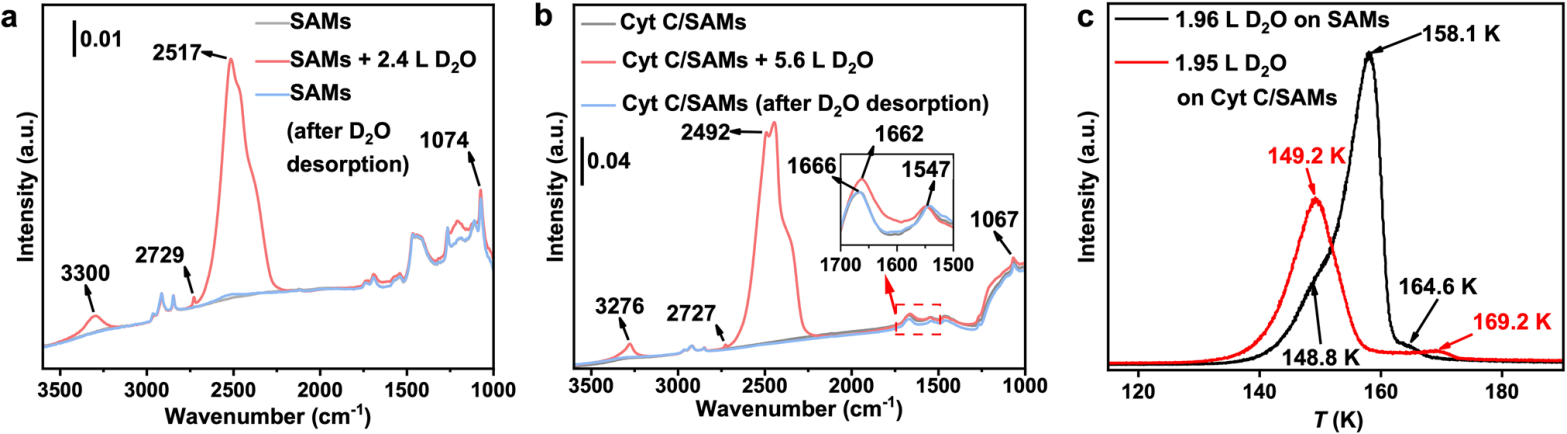
IRRAS spectrum of **a** 2.4 L D_2_O on the SAMs and **b** 5.6 L D_2_O on the Cyt C/SAMs. The water was deposited onto the sample under UHV conditions at sample temperatures of 110 and 115 K, respectively. **c** Temperature-programmed deposition spectra of 2.0 L D_2_O on SAMs (black curve) and Cyt C/SAMs (red curve) deposited at 110 and 115 K, respectively, with a heating rate of 0.05 K s^−1^

To further elucidate the variations in the hydration shell on the surface of the SAMs and the Cyt C/SAMs, we conducted a series of in situ IRRAS measurements to monitor the adsorption and desorption processes of water in these systems. Figure 4a, b presents the IRRAS spectra of the SAMs and the Cyt C/SAMs samples before and after exposure to D_2_O at low temperatures under ultrahigh-vacuum (UHV) conditions. These spectra, acquired using both *p*- and *s*-polarized IR light, provide direct information about the SAMs and Cyt C without needing a reference spectrum. The peaks at 1074 and 1666 cm^−1^ are characteristic vibrational modes of the SAMs (sulfonate groups) [61] and the Cyt C (amide group) [62], respectively (Table S2). The Cyt C amide group is sensitive to water adsorption, displaying a slight redshift of 4 cm^−1^ upon D_2_O adsorption. This shift is reversible upon water desorption, while the SAMs remains intact during water adsorption. The broad peak centered at approximately 2600–2200 cm^−1^ is characteristic of the OD stretching mode [63] of the water (ice) thin film formed on the surfaces of the SAMs and Cyt C/SAMs. In the temperature-programmed desorption (TPD) experiment shown in Fig. 4c, the black curve for the SAMs exhibits a small hump before the desorption, indicating a crystalline phase transition. The red curve for the Cyt C/SAMs lacks a noticeable hump before the desorption peak, suggesting that almost no phase transition occurred. Additionally, a small hump at 169.2 K indicates a second desorption stage. It should be noted that the desorption temperatures of water molecules are sensitive to both the thickness of the water layer and the heating rate. A low heating rate was employed in the UHV desorption experiments due to the poor thermal conductivity of the glass substrate used for preparing the Au (Au^TS^) substrate and the subsequent SAMs and Cyt C/SAMs layers.

**Fig. 5:**
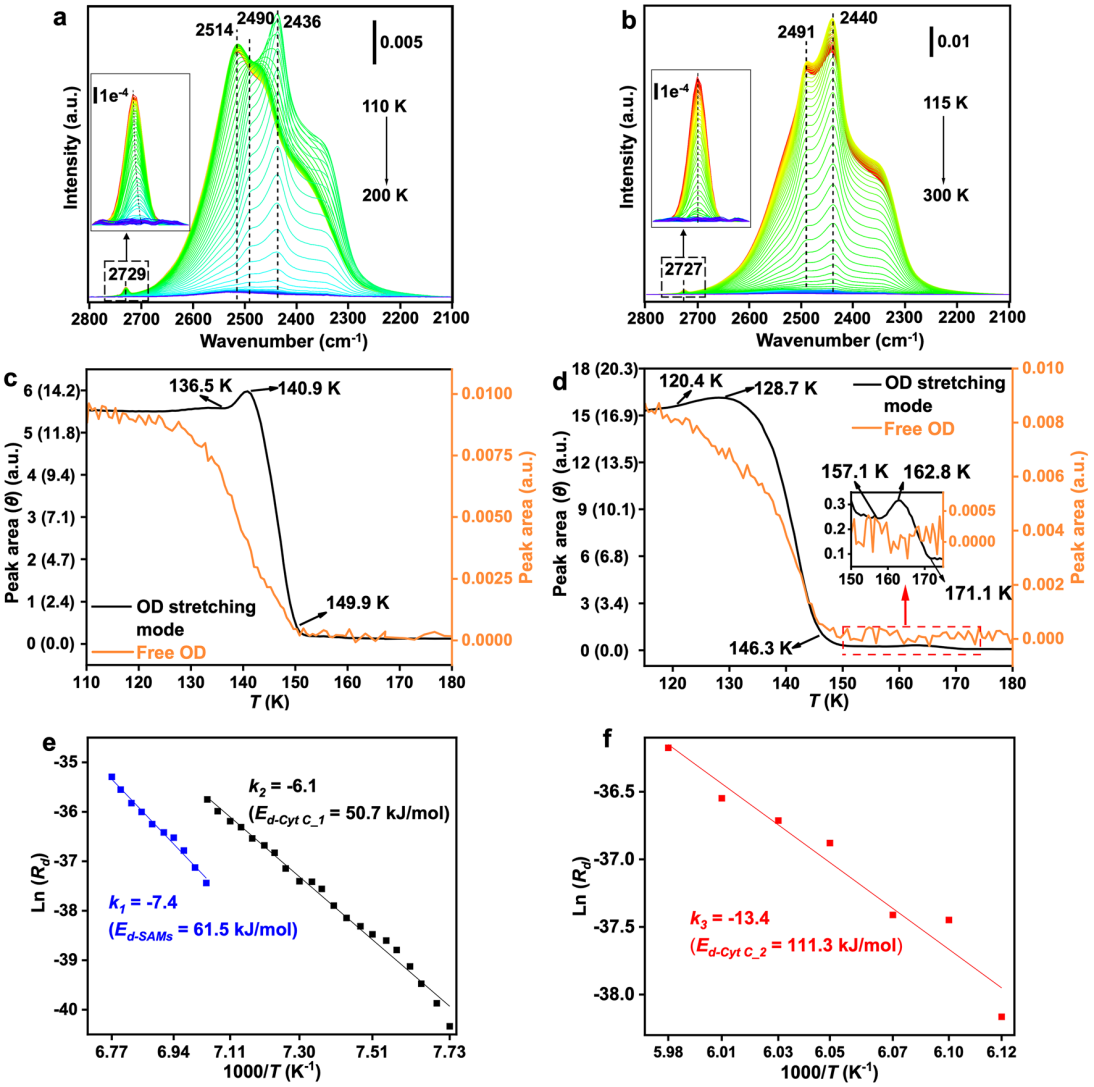
**a**, **b** Temperature-programmed IRRAS spectra recorded as the samples were gradually heated at a rate of 0.01 K s^−1^. **c**, **d** Evolution of the integrated peak area (and the thickness of the water films as denoted by the number of monolayers, θ) of the free OD mode and the OD stretching mode observed in **a** and **b**, respectively, as a function of temperature during annealing. **e**, **f** Activation energies for water desorption from SAMs and Cyt C/SAMs, respectively, determined using the Polanyi–Wigner equation

The position of the main peak is sensitive to the nature of the ice film. For example, the peak at 2517 cm^−1^ observed for the SAMs corresponds to amorphous solid water (ASW). This peak shifts to lower wavenumbers upon a phase transition to crystalline ice (CI) (Fig. 5a). In contrast, the as-deposited ice layer on the Cyt C/SAMs surface exhibits a main peak at 2492 cm^−1^. This may be due to the slightly higher adsorption temperature on Cyt C/SAMs compared to SAMs, which could influence the ASW structure and result in a water layer that is close to the CI phase. Therefore, a less pronounced phase transition to CI was observed (Fig. 5b), consistent with the results shown in Fig. 4c. However, the potential influence of the interaction between Cyt C and water molecules on the properties of the as-deposited ice film cannot be excluded and needs further investigation. It is also worth noting that the weak band at 2729 and 2727 cm^−1^ is attributed to the free OD [63] on the ASW and CI surfaces, respectively (the insets in Fig. 5a, b).

During sample annealing, the water (ice) films begin to desorb from the surface. As shown in Fig. 5c, water is completely desorbed from the SAMs at ~ 150 K, consistent with previous reports [64]. In contrast, two distinct desorption stages were clearly observed for the Cyt C/SAMs (Fig. 5d) [65]. The first desorption region, occurring between 120 and 146 K, may originate from D_2_O desorbing from the SAMs. The second desorption region exhibits an unusually high desorption temperature of approximately 170 K and is attributed to the desorption from the Cty C surface, indicating a strong interaction between the D_2_O and Cyt C (Fig. 5d). Interestingly, a phase transition or structural change (from the ASW phase to the CI phase) occurs in the adsorbed water during the second desorption stage (Fig. S16). The disappearance of the free OD signal from the adsorbed water further demonstrates that the water molecules are strongly coordinated with the Cyt C (Fig. 5d, orange curve).

The activation energy for desorption (*E*_*d*_) can be derived from the Polanyi–Wigner equation [66] (Eq. 1) by analyzing the evolution of the integrated peak area of the OD stretching mode during the desorption processes:

1 $$\ln \left( {R_{d} } \right) = \ln \left( { - \frac{d\theta }{{dT}}} \right) = \ln \left( {\frac{A}{\beta } \theta^{m} } \right) - E_{d} \frac{1}{RT}$$

where* R*_*d*_ denotes the desorption rate,* A* is the frequency factor, *β* (0.01 K s^−1^) is the heating rate and *R* is the ideal gas constant. A first-order process (*m* = 1) can be assumed for simple molecule desorption with a frequency factor of 10^13^ s^−1^. The coverage *θ,* representing the number of adsorbed water layers, is derived from the exposure using the method detailed in Section S4 (Fig. S17). In Fig. 5e, we plot Ln(*R*_*d*_) as a function of 1000/T (K^−1^), where the slope (*k*) is related to the activation energy (*E*_*d*_). A good linear correlation between Ln(*R*_*d*_) and 1000/T was observed, allowing us to extract the activation energy for the SAMs (*E*_*d-SAMs*_) as 61.5 kJ mol^−1^ (from *k*_*1*_ in Fig. 5e, blue curve). For the Cyt C/SAMs, *E*_*d-Cyt_1*_ is 50.7 kJ mol^−1^ for the first desorption stage, likely originating from desorption from the SAMs (extracted from *k*_*2*_ in Fig. 5e, black curve). *E*_*d-Cyt_2*_ is 111.3 kJ mol^−1^ for the second desorption stage, corresponding to desorption from the Cyt C (extracted from *k*_*3*_ in Fig. 5f). Notably, the desorption energy of the Cyt C/SAMs is approximately twice that of the SAMs. By integrating the comprehensive characterizations and analysis of the prepared 2D flakes, it can be concluded that the roles of Cyt C are explained in the following four parts: 1) Since the 2D flakes are composed of both ice-like water and Cyt C, the interaction at the water–SAMs interface is significantly enhanced due to the electrostatic field between the positively charged heme of Cyt C and the negatively charged sulfonate group of the SAMs; 2) the adsorbed Cyt C can act as heterogeneous nucleation sites to promote the nucleation and crystallization of ice-like water [67]; 3) the as-deposited ice layer on the Cyt C/SAMs surface is mainly consisted of the CI phase rather than the ASW phase, and the presence of two distinct desorption regions corresponds to water desorbing from SAMs and Cyt C, respectively. This suggests that the interactions between water and Cyt C are significantly stronger than those between water and metals, where desorption typically occurs below 165 K [64]; 4) the twofold higher *E*_*d-Cyt_2*_ compared to* E*_*d-SAMs*_, indicates that Cyt C can stabilize the 2D flakes under NPT conditions without the need for nanoconfinement.

## Conclusions

In this study, we successfully prepared ice-like 2D flakes with thicknesses varying from ~ 0.7 to ~ 3.3 nm on the SAMs of HSC_11_SO_3_Na through the physisorption and partial desorption of Cyt C under normal pressure and temperature without the need for nanoconfinement. Through comprehensive characterizations, including AFM, nano-AFM-IR, IR spectrum, and Raman spectrum, we identified that these flakes were mainly composed of a few layers of water molecules and the adsorbed Cyt C, exhibiting ice-like properties that were sensitive to temperature and relative humidity. We observed both the melting and the growth processes of these flakes. The melting followed by immediate evaporation process was evidenced by the gradual shrinkage and eventual disappearance of the 2D flakes due to the temperature increase induced by continuous scanning with the AFM tip. Conversely, the growth process was observed by increasing the relative humidity inside the AFM chamber from 60% to 90% and allowing the samples to remain for 10 min. Furthermore, through IRRAS and the related annealing process, we found that the desorption temperature of water from the Cyt C/SAMs (about 171 K) was significantly higher than that from the SAMs (about 146 K). Using the Polanyi–Wigner equation, we determined that the activation energy for water desorption from Cyt C (*E*_*d-Cyt_2*_ = 111.3 kJ mol^−1^) was nearly twice that of the SAMs (*E*_*d-SAMs*_ = 61.5 kJ mol^−1^). This suggests the potential for formation of the ice-like water under ambient conditions without nanoscale confinement on the protein/SAMs monolayers. This work presents an effective method for directly correlating the morphology and composition of the interfacial water on proteins, motivating further investigations into protein–water interactions, the mechanism of ice nucleation on the proteins, and the structural and functional integrity of proteins in the presence of water.

## Supplementary Information

Below is the link to the electronic supplementary material.

Supplementary file1 (DOCX 10900 KB)
