# Supplementary material for: Observation of Ice-Like Two-Dimensional Flakes on Self-Assembled Protein Monolayer without Nanoconfinement under Ambient Conditions
Source: Nanomicro Lett. 2025 Mar 14;17:187. doi: 10.1007/s40820-025-01689-1 (PMC11909351; doi:10.1007/s40820-025-01689-1)
Supplement: Supplementary file 1 — Supplementary file1 (DOCX 10900 KB) [file 40820_2025_1689_MOESM1_ESM.docx]

Supporting Information for

**Observation of Ice-Like** **Two-Dimensional Flakes on Self-Assembled Protein Monolayer without Nanoconfinement under Ambient Conditions**

Wuxian Peng ^1,#^, Linbo Li ^2,#^, Xiyue Bai ^1^, Ping Yi ^2^, Yu Xie ^1^, Lejia Wang ^3^, Wei Du ^4^, Tao Wang ^4^, Jian-Qiang Zhong ^2,^ *^*^*, Yuan Li ^1,^ *^*^*

^1^ Key Laboratory of Organic Optoelectronics and Molecular Engineering and Laboratory of Flexible Electronics Technology, Department of Chemistry, Tsinghua University, Beijing 100084, P. R. China

^2^ School of Physics, Hangzhou Normal University, Hangzhou, Zhejiang 311121, P. R. China

^3^ School of Materials and Chemical Engineering, Ningbo University of Technology, Ningbo, Zhejiang 315211, P. R. China

^4^ Institute of Functional Nano & Soft Materials (FUNSOM), Jiangsu Key Laboratory for Carbon-Based Functional Materials & Devices, Soochow University, Suzhou, Jiangsu 215123, P. R. China

^#^Wuxian Peng and Linbo Li have contributed equally to this work.

***Corresponding authors. E-mail: [zhong@hznu.edu.cn](mailto:zhong@hznu.edu.cn) (Jian-Qiang Zhong); [yuanli_thu@tsinghua.edu.cn](mailto:yuanli_thu@tsinghua.edu.cn) (Yuan Li)

**S1 Mini-discussion about the role of Cyt C and PBS in the formation of the 2D flakes**

In general, the electrostatic interaction between H_2_O and the surface occurs through the electric field near the surface, mainly generated by the sulfonate group of the SAMs. The field strength, in volts per meter (V m^-1^), at distance *z* (perpendicular to the surface) from the center of the ion is given by [S1]：

$$E=\frac{q}{4\pi\varepsilon z^{2}}$$

where *q* is the elementary charge and $\varepsilon$ is the vacuum permittivity. The electric field decays quadratically with distance, and under ambient conditions, it is not strong enough to prevent the suppression of 2D ice formation on SAMs due to strong sublimation caused by the high vapor pressure of water (19.5 Torr) [S2]. G. M. Whitesides *et al*. [S3] reported that the orientations of Cyt C adsorbed on the SAMs is influenced by the surface polarity of SAMs, especially on SAMs of HSC_11_SO_3_^-^, where a hydration layer forms around Cyt C. This hydration layer enhances the electrostatic interaction at the water-SAMs interface, compensating for the entropy reduction during the liquid-solid phase transition of interfacial water [S4]. In addition, the formation of the 2D ice phases depends on the delicate balance between the water-water and water-substrates interactions [S5, S6]. Factors such as the nature of the substrate, the water vapor pressure, the pH and the temperature of the system are critical. Physisorption of Cyt C in pure water may destabilize the protein [S3, S7], which inhibits the formation of the 2D layers. Therefore, no 2D ice-like water was observed in the samples prepared from PBS solution without Cyt C or pure water containing 1mg/ml Cyt C.

**S2** **Fourier Transform infrared (FTIR) Reflectance Measurements**

FTIR measurements were performed using a Bruker Vertex 70 Fourier transform infrared spectrometer in combination with a Hyperion 2000 infrared microscope. A 36×Cassegrain objective (numerical aperture NA = 0.5) was used to collimate the infrared light beam and collect the reflectance spectra. A liquid nitrogen-cooled mercury cadmium telluride detector was used to record the spectra. During the experiments, the knife edge aperture was set to form a measurement area of about 20 × 20 μm^2^. All reflectance spectra were acquired with 1000 scans and a resolution of 4 cm^–1^, in reference to a clean silicon substrate under the same conditions.

**S3 Raman Spectra Characterization**

A confocal Raman microscope (alpha300 R; WITec GmbH, Ulm, Germany) equipped with an SHG Nd: YAG laser (excitation wavelength of 532 nm) and a lens-based spectrometer with a CCD-camera (1600 × 200 pixels) was used to record Raman spectra of the prepared samples. The spectra were collected over a range from 1100 to 4000 cm^−1^ by raster scanning the samples. The spectral resolution was ~3 cm^-1^ owing to the spectrometer’s 600 l/mm grating. The calibration process was performed on a silicon wafer using the most significant mode of silicon at 520 cm^−1^. Confocal Raman measurements were carried out with a 50× Zeiss microscope objective (working distance: 1.1 mm, NA: 0.7). The scan area was 20×20 μm^2^, with Raman imaging parameter set to 200×200 pixels and an integration time of 1 second per pixel.

Raman spectroscopy was used to investigate the vibrational properties of surface water and ice (Fig. S12). Optical images (Fig. S12a, b) show sheet-like structures with a length of approximately 10 μm on the surface of Au^TS^ chips. We measured the Raman spectroscopy of spot #1 in Fig. S12a (away from the sheet-like structure) and spot #2 in Fig. S12b (inside the sheet-like structure) as shown in Fig. S12c, where we focused on the OH-stretching region (2800–3800 cm^−1^) that provided valuable information about water structure [S8]. The broad peak at 3435 cm^−1^ in spectrum #1 was attributed to surface water (the cyan curve in Fig. S12c), while the sharp peak at 3390 cm^−1^ corresponded with the ice-like structure (the orange curve in Fig. S12c) [S9]. Accordingly, the representative Raman mappings recorded at 3435 and 3390 cm^−1^ were shown in the insets of Fig. S12a, b, respectively. These mappings illustrate the intensity distribution of the two Raman peaks in Fig. S12c (water or ice), further identifying the presence of ice-like water on the SAMs after the partial desorption of Cyt C.

**S4 Infrared reflection absorption spectroscopy (IRRAS)**

The coverage *θ* representing the number of adsorbed water layers, is derived from the D_2_O exposures (L), using the equation:

$$Ө=\frac{exposure for uknown coverage}{exposure for known coverage}* known coverage$$

In Fig. S17, the data points can be fitted with two linear functions, intersecting at approximately 0.24 L for SAMs (Fig. S17a) and 0.66 L for Cyt C/SAMs (Fig. S17b). This intersection, assumed to correspond to the completion of the first adsorbed water layer (*θ=*1), was used to calibrate the coverage (*θ*) of adsorbed water layers under the assumption of layer-by-layer growth. This calibration is justified by the typically observed difference in IRRAS absorbance per molecule for the OD stretching mode between the first adsorbed water layer and subsequent layers, a phenomenon attributed to surface selection rules.

**Supplementary Figures and Tables**


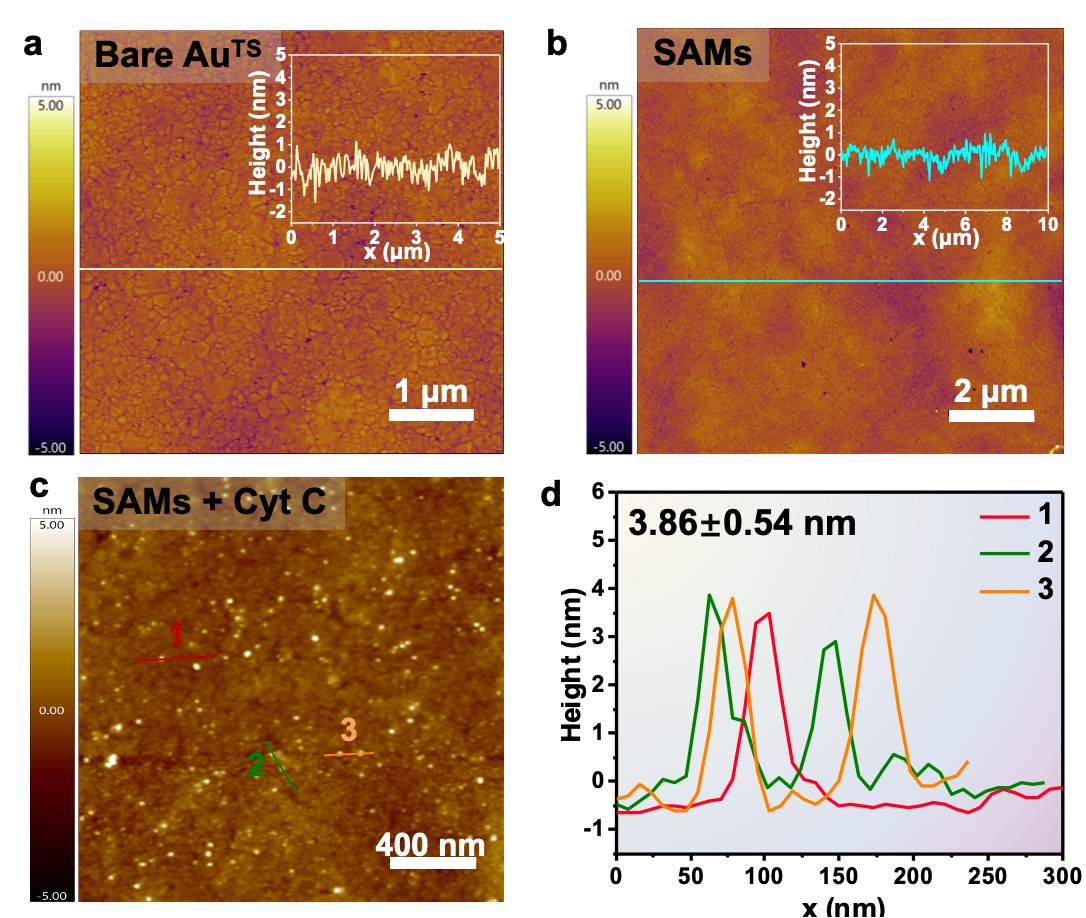


**Fig. S1 a** 2D scans of the Au^TS^ substrate, **b** The SAMs of HSC_11_SO_3_Na, **c** Cyt C absorbed on the surface on SAMs, **d** The height line profiles of the absorbed Cyt C in panel **c**


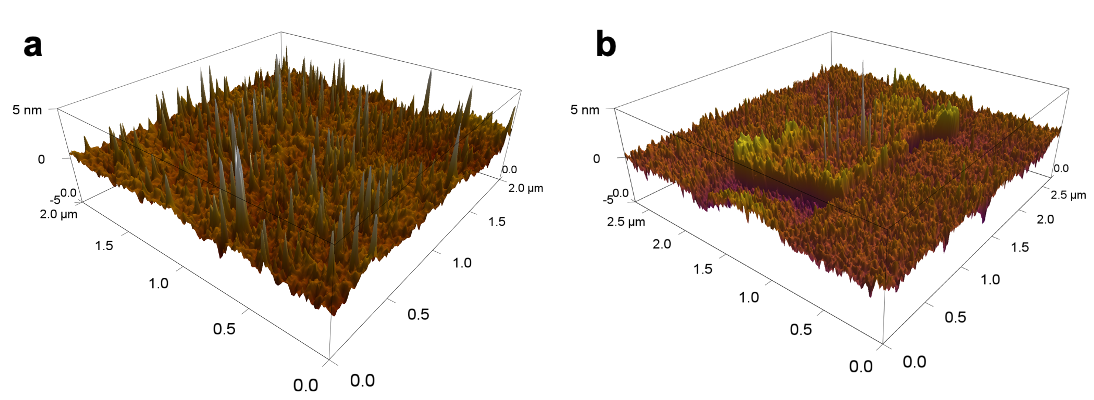


**Fig. S2** 3D AFM images of Cyt C absorbed on SAMs **a** and ice-like water **b**


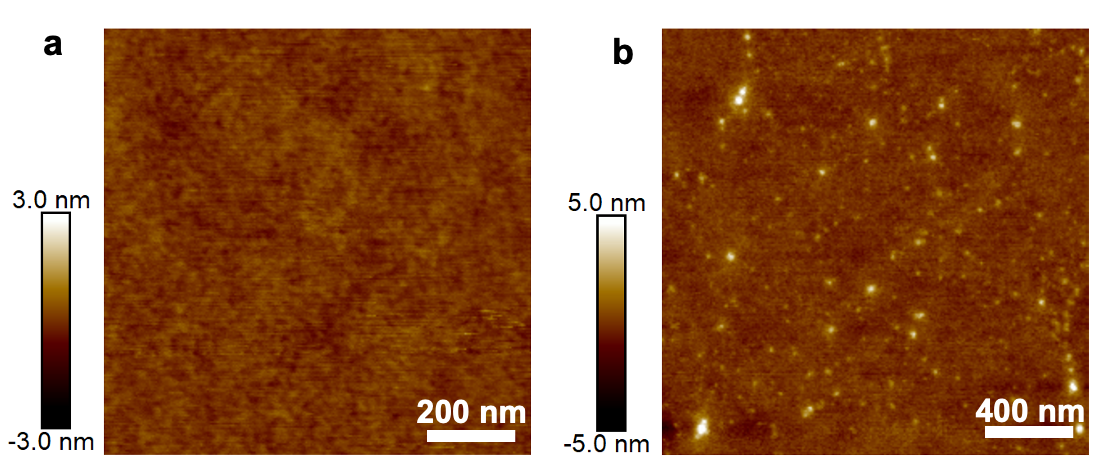


**Fig. S3** **a,** **b** 2D scans of the SAMs of HSC_11_SO_3_Na immersed in PBS without Cyt C and a solution of Cyt C (1 mg/mL) in pure water for 30 mins, respectively, which acted as the control groups of immersion the SAMs in PBS with Cyt C (1 mg/mL)


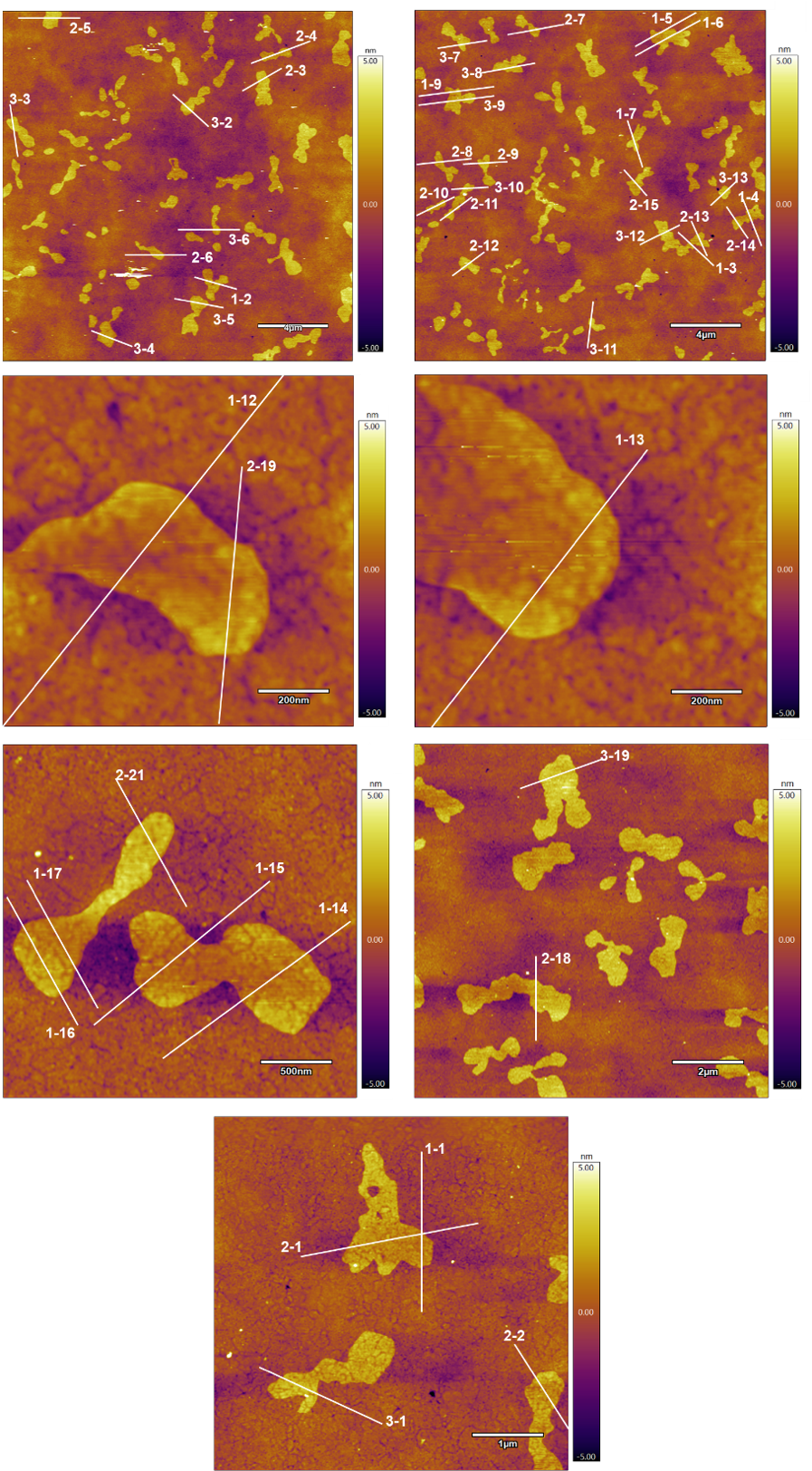


**Fig. S4** 2D scans of the island-like plateaus


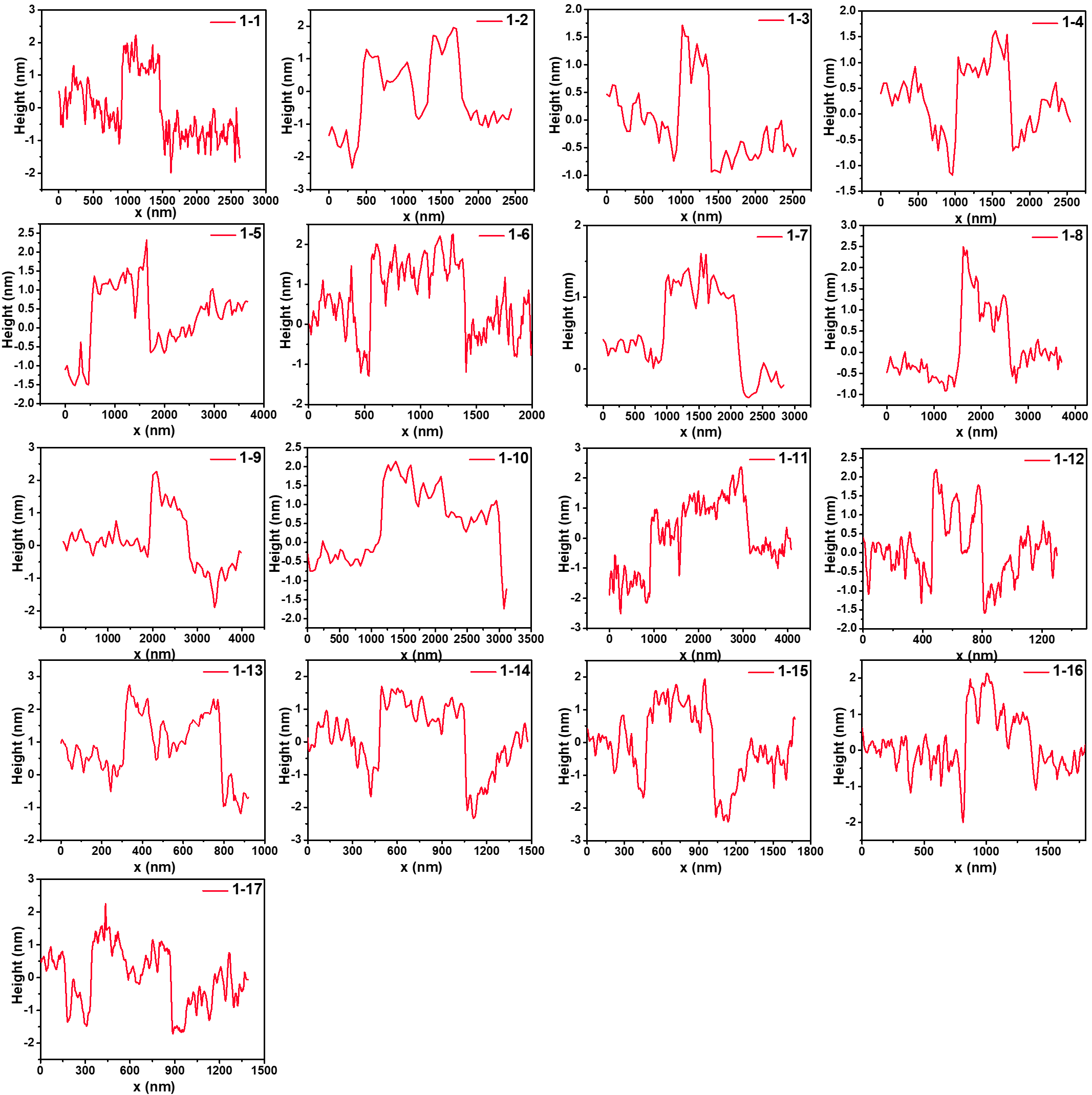


**Fig. S5** Height line profiles of the island-like plateaus with a thickness of approximately 1 nm


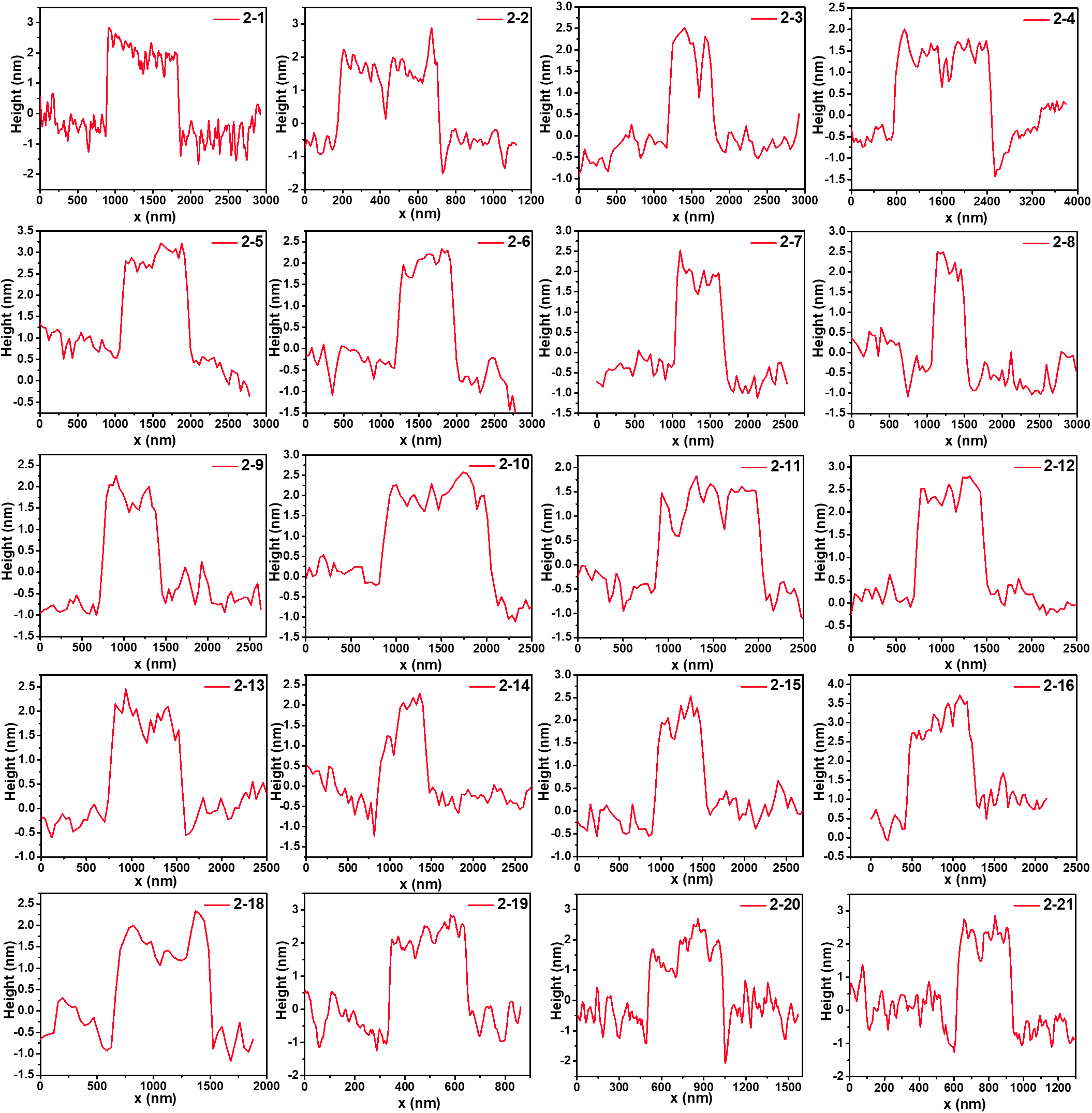


**Fig. S6** Height line profiles of the island-like plateaus with a thickness of approximately 2 nm


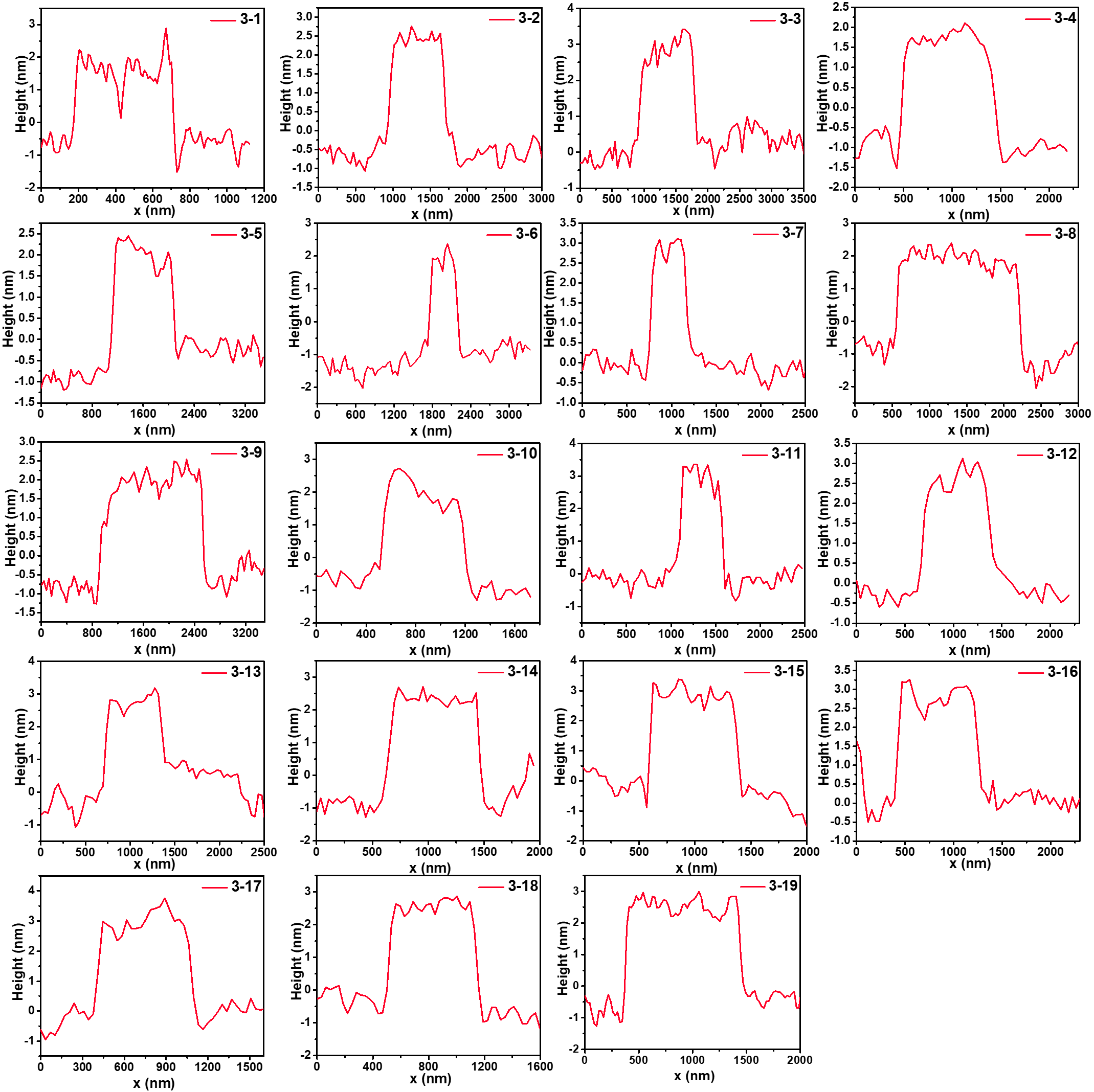


**Fig. S7** Height line profiles of the island-like plateaus with a thickness of approximately 3 nm


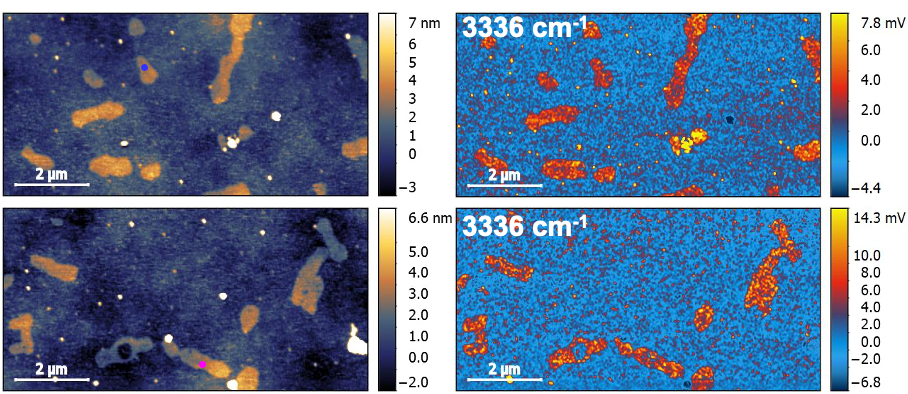


**Fig. S8** AFM-IR height image of the 2D flakes and the related AFM-IR absorption image recorded at 3336 cm^-1^ that corresponded to the distribution of the ice-like water, where the yellow/red color indicated strong absorption and the blue/black color indicated weak or no absorption


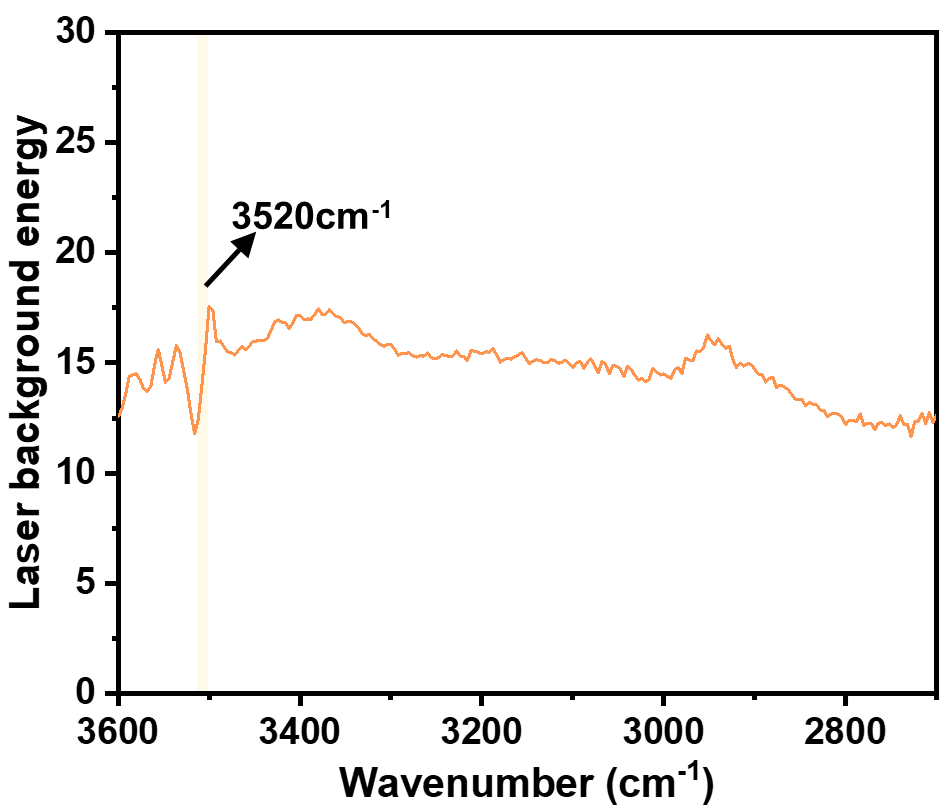


**Fig. S9** The spectrum of the laser background energy of AFM-IR. There is a significant jump in the laser background energy around 3520 cm^-1^, which could lead to the false “peak” in the AFM-IR spectrum and nano-IR images

**
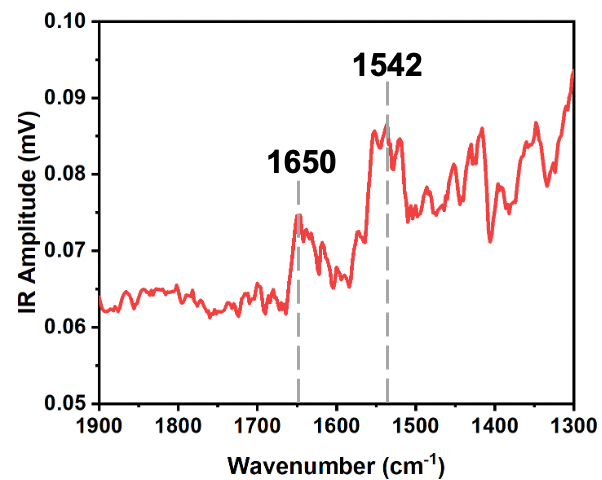
**

**Fig. S10** AFM-IR spectra of spot #1 in panel **b** of **Fig. 2**


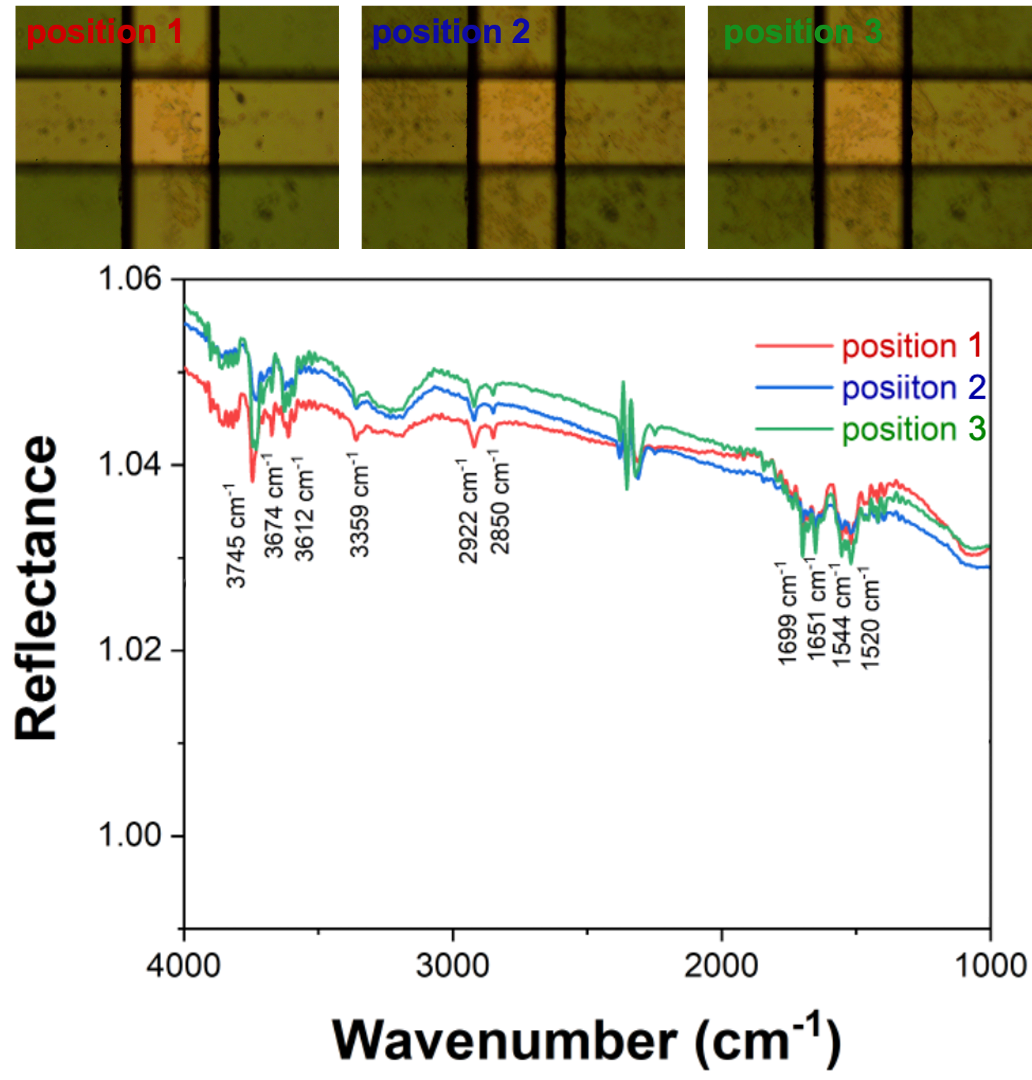


**Fig. S11** The IR spectrum of the 2D flakes on three different positions (the upper panel) carried out at room temperature, RH~60%

**Table S1** IR and Nano-IR spectral interpretations

| Peak | Assignment | Reference number ^a^ |
| --- | --- | --- |
| 1542/1544 cm^-1^ | Amide II | [57] |
| 1650/1651 cm^-1^ | Protein amide I absorption | [57] |
| 1699 cm^-1^ | Amide I [turns and bands] | [57] |
| 2850 cm^-1^ | CH_2_ symmetric | [57] |
| 2922 cm^-1^ | CH stretching | [57] |
| 3336 cm^-1^ | O-H stretching/ N–H stretching | [53-56] |
| 3408 cm^-1^ | O-H stretching | [53-56] |
| 3359 cm^-1^ | O-H stretching | [57] |

^a^ Reference number is the Reference number in the Manuscript


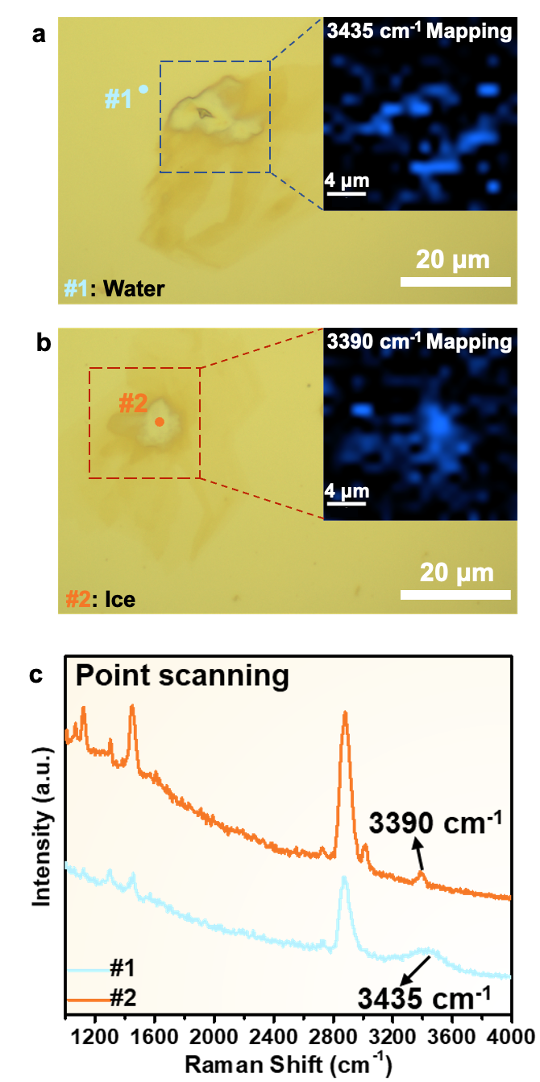


**Fig. S12** Raman spectra of water and ice that adsorbed on the surface after the partial desorption of Cyt C. **a**-**b** The optical images of samples. The insets are the Raman mappings collected at 3435 cm^-1^ and 3390 cm^-1^, respectively. **c** Raman spectrum of spot #1 (cyan) and spot #2 (orange) in panel **a** and **b**. The Raman characterizations were carried out at room temperature, RH~60%


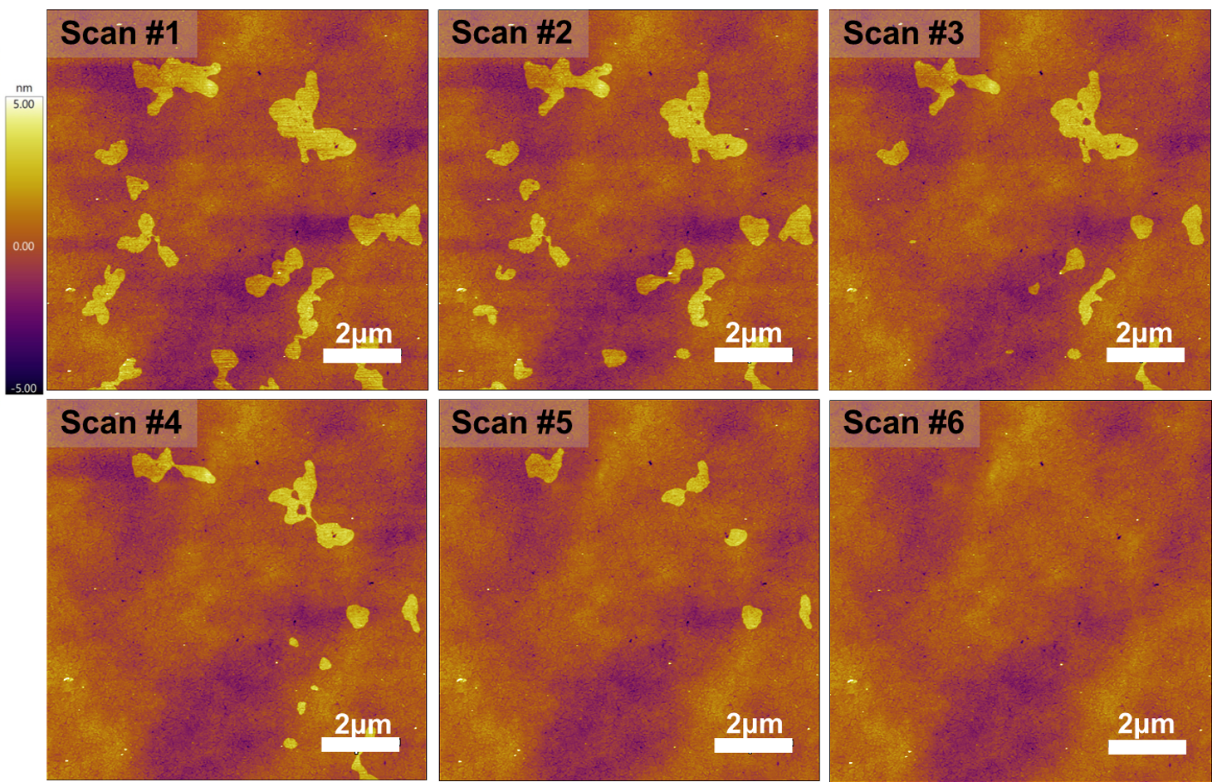


**Fig. S13** The melting followed by the evaporation process of the island-like plateaus by continuous scanning of the AFM tip (room temperature, RH~60%)


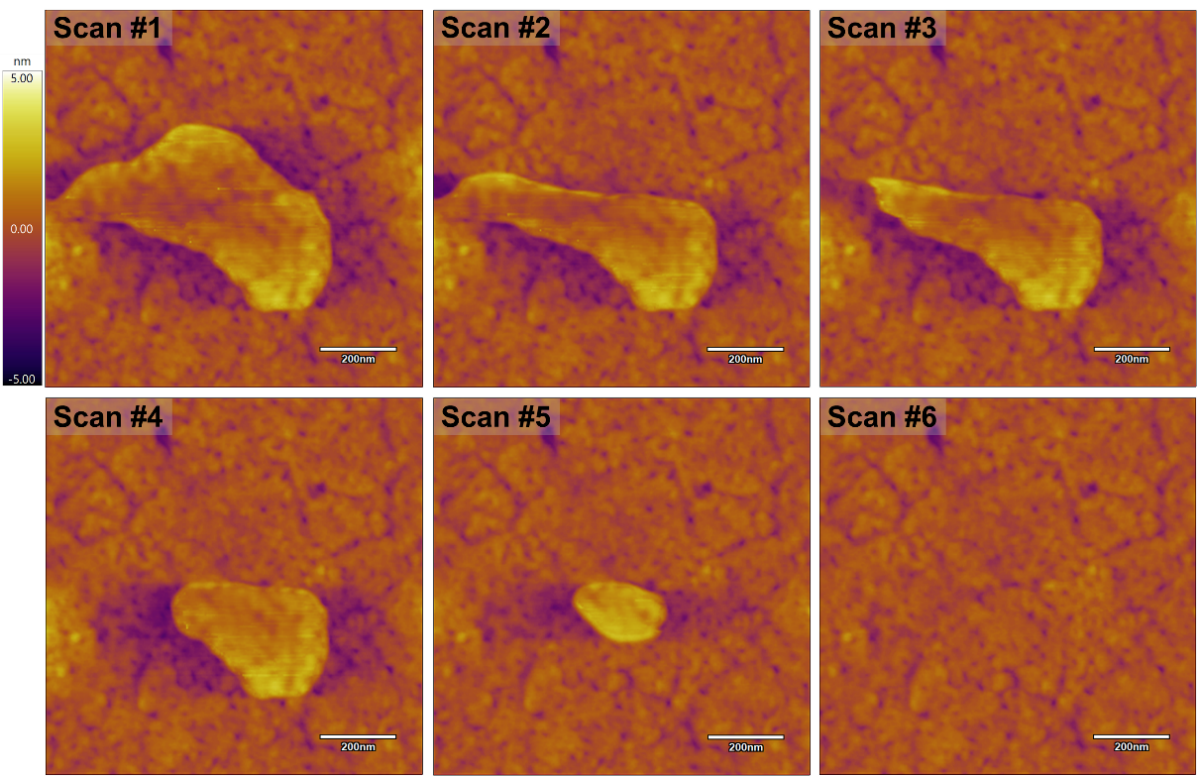


**Fig. S14** The melting followed by the evaporation process of one of the zoomed-in island-like plateaus by continuous scanning of the AFM tip (room temperature, RH~60%)

**
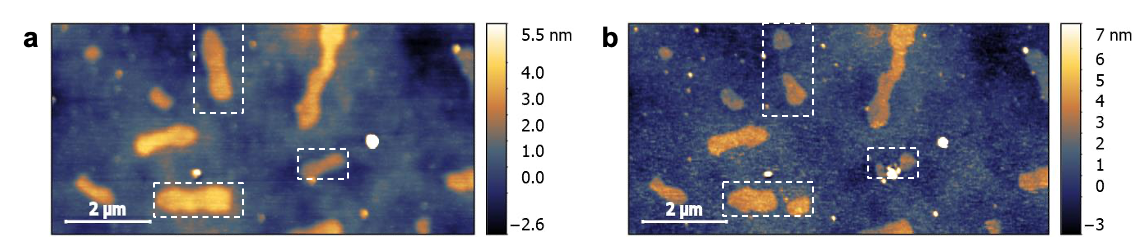
**

**Fig. S15** Nano AFM-IR 2D images before **a** and after **b** continuously scanning

**Table S2** The IRRAS spectral interpretations

| Peak | Assignment | Reference number ^a^ |
| --- | --- | --- |
| 1074 cm^-1^ | SAMs  sulfonate group | [61] |
| 1067 cm^-1^ | Cyt C/SAMs  sulfonate group | [61] |
| 1666 cm^-1^ | Cyt C  Amide I | [62] |
| 2436/2440 cm^-1^ | CI  O-D symmetric stretching mode | [63] |
| 2490 cm^-1^ | CI  O-D asymmetric stretching mode | [63] |
| 2514 cm^-1^ | ASW  O-D asymmetric stretching mode | [63] |
| 2727/2729 cm^-1^ | ASW/CI  free OD | [63] |
| 3276 cm^-1^ | CI  O-H asymmetric stretching mode | [64] |
| 3300 cm^-1^ | ASW  O-H asymmetric stretching mode | [64] |

^a^ Reference number is the Reference number in the Manuscript

**
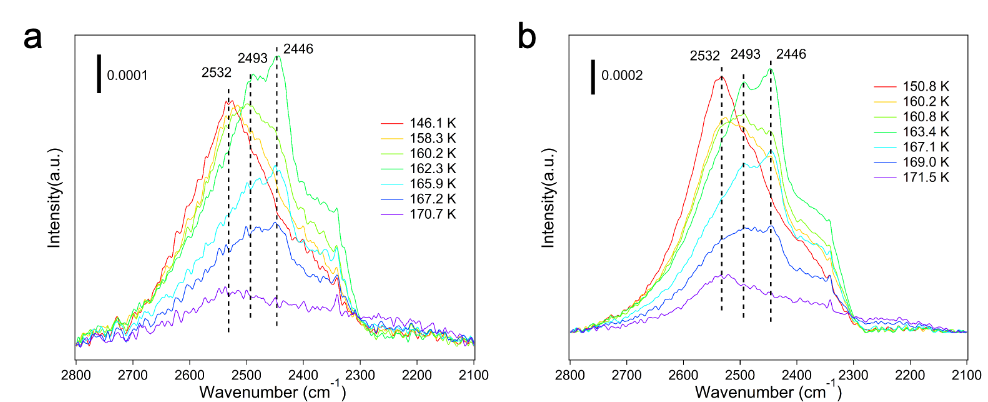
**

**Fig. S16** IRRAS spectra obtained during the second desorption stage of D_2_O on Cyt C/SAMs with exposure of **a** 2.4 L and **b** 5.6 L


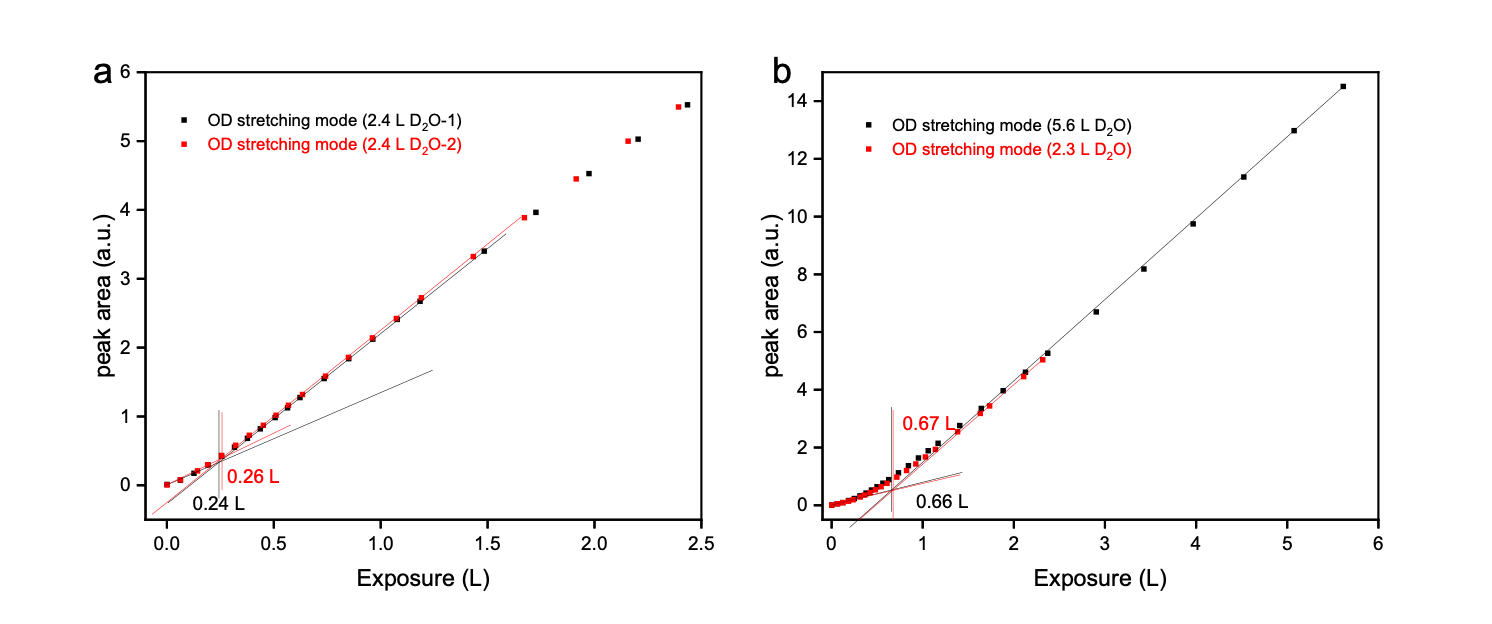


**Fig. S17** The integrated peak area of the OD stretching mode plotted against D_2_O exposures on **a** SAMs at 110 K and **b** Cyt C/SAMs at 115 K

**Supplementary References**

1. G.E. Ewing, Ambient thin film water on insulator surfaces. Chem. Rev. **106**, 1511–1526 (2006). <https://doi.org/10.1021/cr040369x>
2. Y. Zheng, C. Su, J. Lu, K.P. Loh, Room-temperature ice growth on graphite seeded by nano-graphene oxide. Angew. Chem. Int. Ed. **52**, 8708–8712 (2013). <https://doi.org/10.1002/anie.201302608>
3. X. Chen, R. Ferrigno, J. Yang, G.M. Whitesides, Redox properties of Cytochrome*c*Adsorbed on self-assembled monolayers: a probe for protein conformation and orientation. Langmuir **18**, 7009–7015 (2002). <https://doi.org/10.1021/la0204794>
4. C. Zhu, Y. Gao, W. Zhu, J. Jiang, J. Liu et al., Direct observation of 2-dimensional ices on different surfaces near room temperature without confinement. Proc. Natl. Acad. Sci. USA **116**, 16723–16728 (2019). <https://doi.org/10.1073/pnas.1905917116>
5. J. Jiang, Y. Gao, W. Zhu, Y. Liu, C. Zhu et al., First-principles molecular dynamics simulations of the spontaneous freezing transition of 2D water in a nanoslit. J. Am. Chem. Soc. **143**, 8177–8183 (2021). <https://doi.org/10.1021/jacs.1c03243>
6. X. Zhang, J.-Y. Xu, Y.-B. Tu, K. Sun, M.-L. Tao et al., Hexagonal monolayer ice without shared edges. Phys. Rev. Lett. **121**, 256001 (2018). <https://doi.org/10.1103/PhysRevLett.121.256001>
7. M.C. Bellissent-Funel, A. Hassanali, M. Havenith, R. Henchman, P. Pohl et al., Water determines the structure and dynamics of proteins. Chem. Rev. **116**, 7673–7697 (2016). <https://doi.org/10.1021/acs.chemrev.5b00664>
8. X. Xue, Z.-Z. He, J. Liu, Detection of water–ice phase transition based on Raman spectrum. J. Raman Spectrosc. **44**, 1045–1048 (2013). <https://doi.org/10.1002/jrs.4310>
9. E. Maggiore, M. Tortora, B. Rossi, M. Tommasini, P.M. Ossi, UV resonance Raman spectroscopy of weakly hydrogen-bonded water in the liquid phase and on ice and snow surfaces. Phys. Chem. Chem. Phys. **24**, 10499–10505 (2022). <https://doi.org/10.1039/D2CP01072K>
